# Renal Protective Effects of Low Molecular Weight of *Inonotus obliquus* Polysaccharide (LIOP) on HFD/STZ-Induced Nephropathy in Mice

**DOI:** 10.3390/ijms17091535

**Published:** 2016-09-13

**Authors:** Yen-Jung Chou, Wei-Chih Kan, Chieh-Min Chang, Yi-Jen Peng, Hsien-Yi Wang, Wen-Chun Yu, Yu-Hsuan Cheng, Yu-Rou Jhang, Hsia-Wei Liu, Jiunn-Jye Chuu

**Affiliations:** 1 Department of Traditional Chinese Medicine, En Chu Kong Hospital, New Taipei City 237, Taiwan; cyrb61401@gmail.com (Y.-J.C.); jenny7710203@gmail.com (C.-M.C.); 2 Division of Nephrology, Department of Medicine; Chi-Mei Medical Center, Tainan 710, Taiwan; rockiekan@ntu.edu.tw (W.-C.K.); why8@ms61.hinet.net (H.-Y.W.); 3 Department of Biological Science and Technology, Chung Hwa University of Medical Technology, Tainan 717, Taiwan; 4 Department of Pathology, Tri-Service General Hospital, National Defense Medical Center, Taipei 114, Taiwan; yijen0426@gmail.com; 5 Department of Sports Management, College of Leisure and Recreation Management, Chia Nan University of Pharmacy and Science, Tainan 717, Taiwan; 6 Graduate Institute of Life Sciences, National Defense Medical Center, Taipei 114, Taiwan; jushisa1114@gmail.com; 7 Institute of Biotechnology, Southern Taiwan University of Science and Technology, Tainan 710, Taiwan; 4A1H0004@stust.edu.tw (Y.-H.C.); zoe82232@gmail.com (Y.-R.J.); 8 Department of Life Science, Fu Jen Catholic University, New Taipei City 242, Taiwan; 9 Pharmacy, Wei Gong Memorial Hospital, Miaoli 351, Taiwan

**Keywords:** diabetic nephropathy, *Inonotus obliquus*, polysaccharides, renal fibrosis, advanced glycation end products (AGE), NF-κB, TGF-β

## Abstract

Diabetic nephropathy (DN) is the leading cause of end-stage renal disease in diabetes mellitus. Oxidative stress, insulin resistance and pro-inflammatory cytokines have been shown to play an important role in pathogeneses of renal damage on type 2 diabetes mellitus (DM). *Inonotus obliquus* (IO) is a white rot fungus that belongs to the family Hymenochaetaceae; it has been used as an edible mushroom and exhibits many biological activities including anti-tumor, anti-oxidant, anti-inflammatory and anti-hyperglycemic properties. Especially the water-soluble *Inonotus obliquus* polysaccharides (IOPs) have been previously reported to significantly inhibit LPS-induced inflammatory cytokines in mice and protect from streptozotocin (STZ)-induced diabetic rats. In order to identify the nephroprotective effects of low molecular weight of IOP fraction (LIOP), from the fruiting bodies of *Inonotus obliquus*, high-fat diet (HFD) plus STZ-induced type 2-like diabetic nephropathy C57BL/6 mice were investigated in this study. Our data showed that eight weeks of administration of 10–100 kDa, LIOP (300 mg/kg) had progressively increased their sensitivity to glucose (less insulin tolerance), reduced triglyceride levels, elevated the HDL/LDL ratio and decreased urinary albumin/creatinine ratio(ACR) compared to the control group. By pathological and immunohistochemical examinations, it was indicated that LIOP can restore the integrity of the glomerular capsules and increase the numbers of glomerular mesangial cells, associated with decreased expression of TGF-β on renal cortex in mice. Consistently, three days of LIOP (100 μg/mL) incubation also provided protection against STZ + AGEs-induced glucotoxicity in renal tubular cells (LLC-PK1), while the levels of NF-κB and TGF-β expression significantly decreased in a dose-dependent manner. Our findings demonstrate that LIOP treatment could ameliorate glucolipotoxicity-induced renal fibrosis, possibly partly via the inhibition of NF-κB/TGF-β1 signaling pathway in diabetic nephropathy mice.

## 1. Introduction

Diabetic mellitus (DM) is the most serious chronic disease in the world, and approximately 90% of diabetic patients belong to type 2 DM [1]. Moreover, a common complication confronted by diabetic patients, diabetic nephropathy (DN), is the most important cause of death in insulin-dependent diabetes mellitus, as 30%–45% of patients eventually develop end-stage renal failure [2]. Hyperglycemia leads to elevation of oxidative stress, insulin resistance and pro-inflammatory cytokines, which are the key pathogeneses of renal damage in type 2 DM [3,4].

*Inonotus oblique* is a white rot fungus that belongs to the family *Hymenochaetaceae* (of Basidiomycetes) that is widely distributed in Russia, North America, Europe, Japan and northeastern China [5]; it has been used as an edible mushroom without any adverse side effects in treatment of cancers and digestive system diseases [6,7]. In recent years, it has been reported that *Inonotus obliquus* extracts have various biological activities, such as anti-inflammatory, anti-nociceptive and anti-hyperglycemic properties [8,9]. Mushroom polysaccharides (e.g., *Inonotus obliquus*) are considered to be one of the major active substances because they have exhibited many biological activities including anti-tumor, anti-oxidant, hypoglycemic and immune-stimulating effects [10,11,12].

*Inonotus obliquus* polysaccharides (IOP), especially the extracellular polysaccharides (EPS), had significant hydroxyl and 2,2-diphenyl-1-picrylhydrazyl (DPPH) radical-scavenging activity while the low molecular weight (29 kDa) had higher anti-oxidant activity [13]. It was shown that the water-soluble IOP fraction could inhibit LPS-induced inflammatory cytokines, IL-1β, TNF-α and IL-6, showing a dose-dependent anti-inflammatory effect in a RAW 264.7 macrophage [14]. Accordingly, a novel water-soluble IOP, named IP3a, with an average molecular weight of 48 kDa could promote cytokine secretion (IL-2, IL-6, IL-12 and TNF-α) and macrophage phagocytosis in mice [15]. Further study indicated that IOP is effective in the protection of STZ-induced diabetic rats [16]; especially IOP with a molecular weight of 32.5 kDa are capable of alleviating pancreatic acinar atrophy in diethyldithiocarbamate (DDC)-induced mice [17]. For a pharmacological study of diabetic kidney disease in response to mushroom polysaccharide therapy, a water-soluble polysaccharide (CPS-2) isolated from the cultured *Cordyceps sinensis*, with a molecular weight of 43.9 kDa, could significantly relieve chronic renal disease by lowering blood urea nitrogen and serum creatinine [18]. *Ganoderma lucidum* polysaccharides (GL-PS) were also reported as potentially reducing serum glucose, creatinine (Cr), blood urea nitrogen (BUN), triglyceride (TG), urinary albumin excretion (UAE) and eventually improving the renal morphometric changes (glomerular size and mesangial matrix) on STZ-induced diabetic nephropathy in mice [19].

Accordingly, mice fed with a high-fat diet (HFD), followed by a single intraperitoneal injection of streptozotocin, have been successfully used to develop an optimal diabetic nephropathy (DN) model, which may be a result of the dyslipidemia followed by inflammation, oxidative stress, insulin resistance and renal fibrosis [20]. However, few studies have focused on the therapeutic effects of IOP extracts/fractions from submerged culture [21,22], and little information is available about IOP characteristics from the fruiting bodies of *Inonotus obliquus* and the nephroprotective effects in high fat diet-induced diabetic nephropathy mice. Currently, several studies have presented that NF-κB is a key transcription factor for excessive inflammatory responses mediating the development and progression of diabetic nephropathy [23]. Hyperglycemia induces NF-κB activation, leading to increased transforming growth factor β (TGF-β); TGF-β-dependent signaling in turn facilitates fibrosis and suppresses inflammation that characterize diabetic nephropathy (DN) [24].

Advanced glycation end products (AGEs) are assumed to play a key role in diabetic nephropathy (DN) [25]. AGEs have been shown to exert marked expression of TGF-β 1 in LLC-PK1, resulting in a significant increase in DNA damage and marked elevation in renal insufficiency [26]. It has also been proposed that AGEs reduce protein breakdown as a result of decreased lysosomal proteinase activities following a series of protein synthesis, eventually causing hypertrophy of LLC-PK1 cells [27]. In cultured glomerular mesangial cells (GMCs), NF-κB-regulated inflammatory factors TGF-β1 in high glucose-treated GMCs caused significant accumulation of fibronectin (FN), playing an important role in diabetic renal fibrosis. Stark increases were consistent with the decrease of cell proliferation in high glucose-treated GMCs, leading to accumulation of fibronectin (FN), an important indicator of renal fibrosis [28]. Accumulating evidence indicates that several herbal therapeutic agents and natural products exhibited renal protective effect in diabetic rats partly through anti-hyperglycemia, which was accompanied by attenuation of inflammatory processes via inhibition of NF-κB/TGF-β1 signaling pathway in streptozotocin-induced diabetic nephropathy rat model [29,30].

In order to identify the active nephroprotective components of IOP, the ameliorating effects of low molecular weight of IOP fraction (LIOP) by hot water extraction on hyperglycemia, hyperinsulina and hypercholesterolemia occurred on high-fat diet (HFD) plus streptozotocin (STZ)-induced type 2-like diabetic nephropathy C57BL/6 mice were investigated. Also, we explore whether LIOP was linked to altered AGE-mediated renal tubulointerstitial fibrosis in DN, to examine the possible mechanisms of LIOP responsible for inhibition of AGE-induced cell death in renal tubular epithelial cells (LLC-PK1, a pig kidney proximal tubules cell line) in vitro. Thus, the purpose is to focus on the anti-fibrosis properties of water-soluble LIOP fractions for searching new natural ingredients used in functional food and the pharmaceutical industry to alleviate the diabetic nephropathy.

## 2. Results

### 2.1. Inonotus obliquus (IO) Polysaccharide Analysis by Gel Permeation Chromatography (GPC)

**Figure 1:**
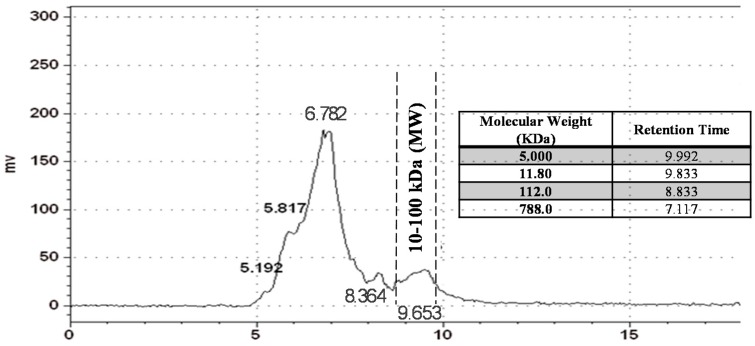
Profile of the molecular weight distribution of crude IOP extract by gel permeation chromatography analysis.

As shown in Figure 1, the total yield of crude water-soluble polysaccharide from the dried fruiting bodies of *Inonotus oblique* (IO) by hot water extraction reached the maximum value (15.8%) based on the starting amount by phenol-sulphuric acid method when extraction temperature was 100 °C (Data not shown). On the basis of the equation determined by calculation of linear regression of standard dextrans, the molecular weight distribution of crude polysaccharide extract was determined by gel-permeation chromatography (GPC), showing that it was eluted as two relative wide group peaks’ molecular weight, estimated to be >1000 kDa and 10–100 kDa, respectively, as determined by GPC chromatogram. By GPC profiles of crude water-soluble polysaccharide from IO, we use the disposable ultrafiltration device (Vivaspin^®^ 500) to obtain the low-molecular-weight, 10–100 kDa (within the dotted line range) *Inonotus oblique* polysaccharide (LIOP), by fractions of a cut-off value (10, 30, 50 and 100 kDa) then subsequently approach the animal tests and cellular assays.

### 2.2. Effect of Inonotus obliquus Polysaccharides (LIOP) on the Glucose Tolerance Test (OGTT) in C57BL/6 Mice

**Figure 2:**
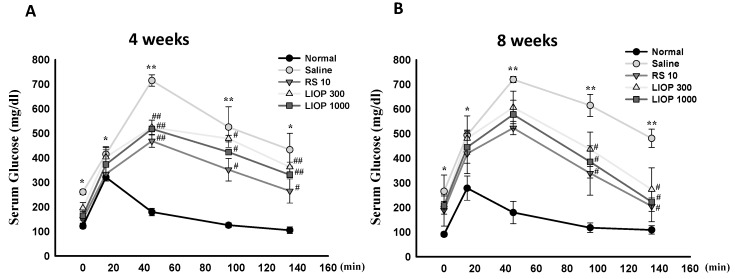
OGTT Patterns of LIOP. After an overnight fast, 3 g/kg of d-glucose was administered orally to C57BL/6 mice with HFD + STZ-induced diabetic nephropathy. The effects of LIOP (300 and 1000 mg/kg) and rosiglitazone (10 mg/kg) treatment were compared to the effects of the saline-only group at four (**A**) and eight weeks (**B**). The blood samples were taken at 0, 15, 45, 95, and 135 min after administration of d-glucose, and glucose levels were measured in whole venous blood with a glucometer (One Touch II, Lifescan). The data are expressed as mean ± SE of eight mice. *: *p* < 0.05, **: *p* < 0.001 compared to normal group. #: *p* < 0.05, ##: *p* < 0.001 compared to saline group. OGTT: oral glucose tolerance test; Normal: native control; Saline: vehicle control; RS: rosiglitazone; LIOP: Low-molecular-weight, 10–100 kDa polysaccharides from *Inonotus obliquus* extract.

For the AUC (area under the curve) patterns of the OGTTs, the OGTT results of C57BL/6 mice fed HFD (40% fat) + STZ (50 mg/Kg) showed non-fasting blood glucose over 150 mg/dL (data not shown) and a large AUC compared with the normal group (fed a normal diet) at four weeks (Figure 2A) and eight weeks (Figure 2B), respectively. The profiles of the curves of the plots showed that the AUC curves in LIOP-treated (300 and 1000 mg/kg) or RS (rosiglitazone, 10 mg/kg)-treated mice at four weeks (Figure 2A) and eight weeks (Figure 2B) were significantly smaller than those of the saline-treated mice. Our data showed that mice in the LIOP groups (300 and 1000 mg/kg) and RS group progressively increased in their sensitivity to glucose (approximately returned to the starting base level) as compared to the saline group at eight weeks; however, the difference in glucose tolerance between the LIOP groups (300 and 1000 mg/kg) and RS (10 mg/kg) group were not apparent as compared with the saline group at four weeks throughout the study period, at 0, 15, 45, 95 and 135 min.

### 2.3. Effect of LIOP on the Insulin Tolerance Test (ITT) in C57BL/6 Mice

**Figure 3:**
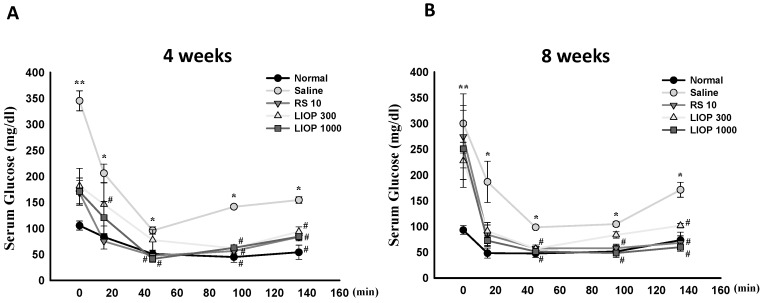
ITT Patterns of LIOP. After an overnight fast, 0.5 U insulin was administered orally to C57BL/6 mice with HFD + STZ-induced diabetic nephropathy. The effects of LIOP (300 and 1000 mg/kg) and rosiglitazone (10 mg/kg) treatment were compared to the effects of the saline-alone group at four (**A**) and eight weeks (**B**). The blood samples were taken 0, 15, 45, 95, and 135 min after administration of insulin, and glucose levels were measured in whole venous blood with a glucometer (One Touch II, Lifescan). The data are expressed as mean ± SE of eight mice. *: *p* < 0.05, **: *p* < 0.001 compared to normal group. #: *p* < 0.05, compared to saline group. ITT: insulin tolerance test; Normal: native control; Saline: vehicle control; RS: rosiglitazone; LIOP: Low-molecular-weight, 10–100 kDa polysaccharides from *Inonotus obliquus* extract.

After an insulin injection, the C57BL/6 mice fed HFD (40% fat) + STZ (50 mg/Kg) revealed elevations in fasting blood glucose levels over that of mice fed a normal diet during an all-time points test. Accordingly, the ITT profiles also showed that the AUC curves in LIOP-treated (300 and 1000 mg/kg) or RS (rosiglitazone, 10 mg/kg)-treated mice were relatively smaller than those of the saline-treated mice at four weeks (Figure 3A) and eight weeks (Figure 3B), respectively. Our results indicated that either LIOP groups (300 and 1000 mg/kg) or the RS (10 mg/kg) group significantly decreased fasting blood glucose levels and had less insulin tolerance compared to that of the saline group at four and eight weeks, throughout the study period, at 0, 15, 45, 95 and 135 min.

### 2.4. Effects of LIOP on the Insulin Concentration and Triglyceride, Total Cholesterol and HDL/LDL Ratio in C57BL/6 Mice

**Figure 4:**
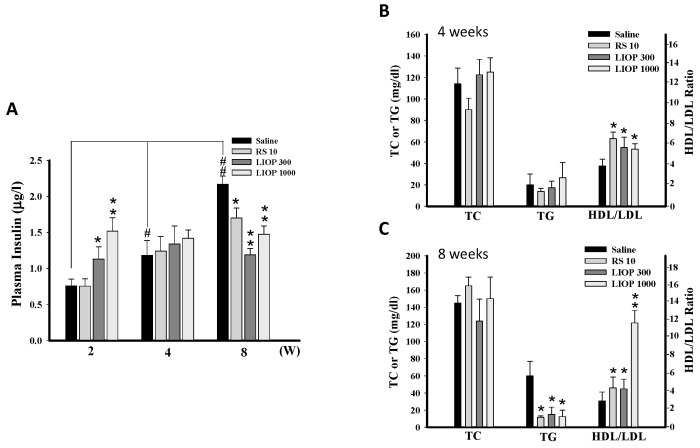
Plasma insulin levels and serum lipid profile. Blood samples were collected from the retro-orbital sinus of C57BL/6 mice with HFD + STZ-induced diabetic nephropathy. The plasma insulin levels were measured by ELISA at two, four, and eight weeks (**A**) and the plasma triglyceride (TG), total cholesterol (TC) and HDL/LDL ratio, respectively, were determined by the biochemistry analyzers at four and eight weeks. The modulation of plasma glucose and lipid metabolism were measured following LIOP (300 and 1000 mg/kg) and rosiglitazone (10 mg/kg) treatment as compared to the effects of the saline-alone group at four (**B**) and eight weeks (**C**). The data are expressed as mean ± SE of eight mice. *: *p* < 0.05, **: *p* < 0.01 as compared with the saline group at two, four, eight weeks. #: *p* < 0.05, ##: *p* < 0.01 compared with saline group at the two-week trial. Normal: native control; Saline: vehicle control; RS: rosiglitazone; LIOP: Low-molecular-weight, 10–100 kDa polysaccharides from *Inonotus obliquus* extract.

Plasma insulin levels were time-dependently higher in the saline-treated in C57BL/6 mice fed HFD (40% fat) + STZ (50 mg/Kg). In contrast to RS (rosiglitazone, 10 mg/kg), LIOP (300 and 1000 mg/kg) treatment can increase insulin level at two weeks (*p* < 0.05 and *p* < 0.01, respectively) but decrease insulin level at eight weeks compared to the saline-treated group (*p* < 0.01 and *p* < 0.01, respectively) (Figure 4A). Despite consecutive four-weekw administration of LIOP (300 and 1000 mg/kg), there was no obvious effect on cholesterol (*p* > 0.05 and *p* > 0.05, respectively) and triglyceride (*p* > 0.05 and *p* > 0.05, respectively) levels compared to saline group (Figure 4B), but consecutive eight-week administration of LIOP (300 and 1000 mg/kg) could significant reduce triglyceride levels in C57BL/6 mice (*p* < 0.05 and *p* < 0.05, respectively) (Figure 4C). Interestingly, four- and eight-week administration of LIOP (300 and 1000 mg/kg) are similar to RS treatment and significantly elevated the HDL/LDL ratio compared to the saline group (*p* < 0.05 and *p* < 0.05, respectively at four weeks and *p* < 0.05 and *p* < 0.01, respectively, at eight weeks).

### 2.5. Effect of LIOP on Kidney Function in C57BL/6 Mice

**Figure 5:**
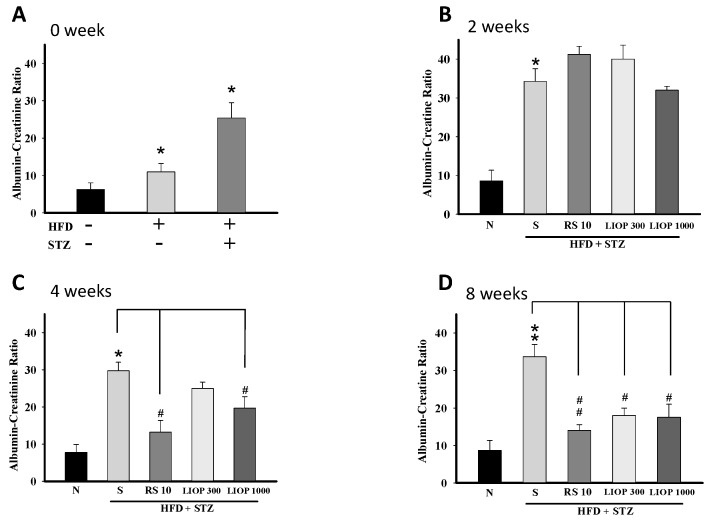
Urine albumin-creatinine ratio (ACR). Urine samples were collected from C57BL/6 mice with HFD + STZ-induced diabetic nephropathy by the receiver of metabolic cage. The urine albumin and creatinine levels were measured by the biochemistry analyzers and the albumin-creatinine ratio (ACR) were calculated. First, the ACR values were analyzed after a twi-month induction phase on HFD alone-induced, STZ-induced alone and HFD + STZ-induced C57BL/6 mice, respectively (**A**). The change of ACR values were also detected following LIOP (300 and 1000 mg/kg) and rosiglitazone (10 mg/kg) treatment as compared to the effects of the normal group and saline group, alternatively at two (**B**); four (**C**) and eight weeks (**D**). The data are expressed as mean ± SE of eight mice. *: *p* < 0.05, **: *p* < 0.01 compared with the normal group. #: *p* < 0.05, ##: *p* < 0.01 compared with the saline group. Normal: native control; Saline: vehicle control; RS: rosiglitazone; LIOP: Low-molecular-weight, 10–100 kDa polysaccharides from *Inonotus obliquus* extract.

Overt kidney damage and filtration impairment can be manifested at percent change by elevated blood creatinine levels and albumin leakage. After a two-month regiment, a significant elevation in urinary ACR was observed for the C57BL/6 mice treated with STZ (50 mg/Kg) alone or HFD (40% fat) + STZ (50 mg/Kg) compared to normal C57BL/6 mice (Figure 5A). At two weeks, there was no significant difference in urinary ACR between the LIOP (300 and 1000 mg/kg) and RS (rosiglitazone, 10 mg/kg) groups compared to the saline group in C57BL/6 mice (*p* > 0.05, *p* > 0.05 and *p* > 0.05, respectively) (Figure 5B). However, urinary ACR was significantly decreased in LIOP (1000 mg/kg)-treated and RS (rosiglitazone, 10 mg/kg)-treated C57BL/6 mice compared with saline-treated C57BL/6 mice at four weeks (*p* < 0.05 and *p* < 0.05, respectively) (Figure 5C) and eight weeks (*p* < 0.05 and *p* < 0.01, respectively) (Figure 5D).

### 2.6. Renal Hematoxylin and Eosin and Immunohistochemical Staining in the C57BL/6 Mice

**Figure 6:**
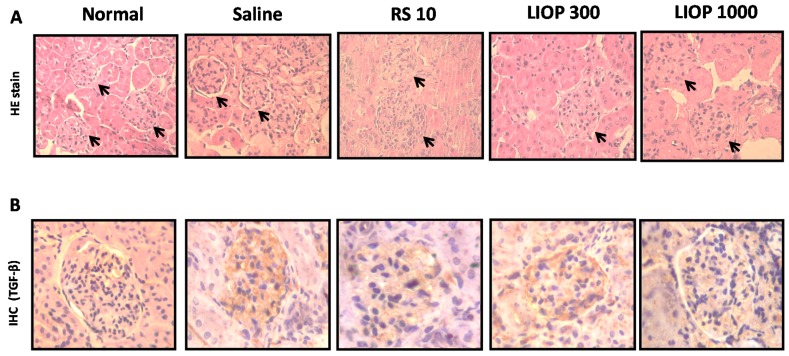
Morphological and immunohistochemical examination. Histopathological study of the renal cortex in C57BL/6 mice with HFD + STZ-induced diabetic nephropathy was examined after LIOP (300 and 1000 mg/kg) and rosiglitazone (10 mg/kg) treatment as compared to the effects of the saline group, alternatively at eight weeks. In the normal group, focal glomerulus (**black arrow**) remained relatively intact and numerous with hematoxylin and eosin staining (**A**). Original magnification: ×100. Representative renal cortex sections with immunostained TGF-β (in **brown color**) were shown as in glomerulus area treated with LIOP (300 and 1000 mg/kg) and rosiglitazone (10 mg/kg) at eight weeks (**B**). Original magnification: ×400. Normal: native control; Saline: vehicle control; RS: rosiglitazone; LIOP: Low-molecular-weight, 10–100 kDa polysaccharides from *Inonotus obliquus* extract.

At eight weeks, renal cortexes of mice were examined using hematoxylin and eosin (H & E) to evaluate the severity of overt nephropathy as indicated by segmental expansion of the mesangium and collagen fibril deposition in glomeruli. As shown by H & E stain in Figure 6A, the renal cortex of native C57BL/6 mice (control group) who were fed a normal diet showed relatively intact glomeruli with numerous nuclei, but the C57BL/6 mice fed HFD (40% fat) + STZ (50 mg/Kg) enhanced the mesangial matrix accumulation and increased in the fractional volume of mesangium per glomerulus. Samples from mice treated with LIOP (300 and 1000 mg/kg) similar to RS (rosiglitazone, 10 mg/kg), can restore the integrity of the glomerular capsules and increase the numbers of glomerular mesangial cells compared to the saline group in renal cortex, suggesting that LIOP might attenuate the degree of segmental expansion of the mesangium and mesangial matrix accumulation on renal cortex. The average of numbers of the nucleus in the glomerulus area were shown as 51 ± 15 (normal), 32 ± 9 (saline), 52 ± 10 (RS), 49 ± 7 (LIOP, 300 mg/kg) and 54 ± 10 (LIOP, 1000 mg/kg), respectively. Consistently, the TGF-β1 protein expression was significantly higher in the C57BL/6 mice fed HFD (40% fat) + STZ (50 mg/Kg) compared with the native C57BL/6 mice (normal group). Meanwhile, it was noted that LIOP (1000 mg/kg) rather than LIOP (300 mg/kg) or RS (rosiglitazone, 10 mg/kg), reduced TGF-β1 protein expression in glomerular of C57BL/6 mice compared with the saline group (*p* < 0.01, *p* < 0.05 and *p* < 0.05, respectively) (Figure 6B). The percentage of TGF-β-positive cells by the total number of nuclei in the glomerulus area in the following order: Normal (9% ± 2%), Saline (76% ± 11%), RS (42% ± 12%), 300 mg/kg LIOP (39% ± 8%) and 1000 mg/kg LIOP (22% ± 5%). Our results indicated that LIOP attenuate renal fibrosis-related mesangial cell proliferation and mesangial matrix accumulation through the inhibition of TGF-β in kidney.

### 2.7. The Protective Effect of LIOP in STZ + AGEs-Induced Renal Cell Toxicity

**Figure 7:**
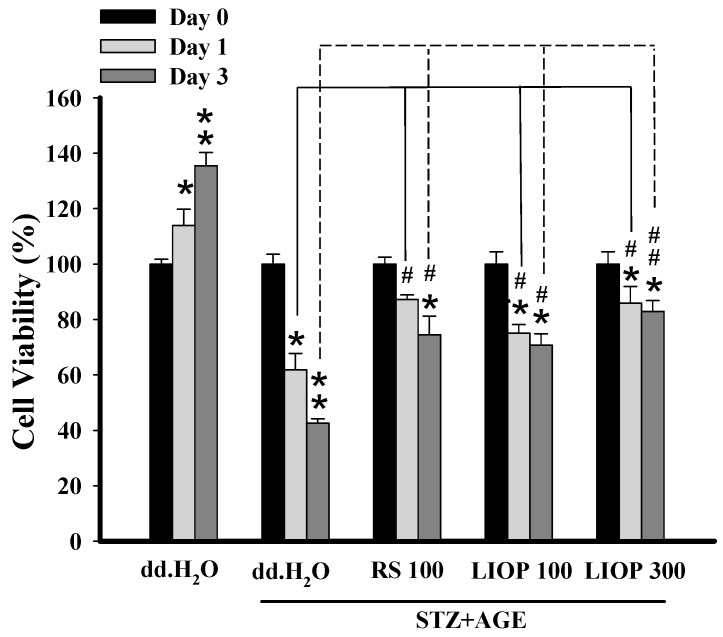
The protection from STZ + AGEs-induced cell toxicity. The renal tubular epithelial cells (LLC-PK1) in vitro were treated with STZ (8 mM) + AGEs (3 mg/mL) causing cell gluctotoxicity at 24 and 72 h. Co-treatment of LIOP (100 and 300 μg/mL) and RS (100 μg/mL), the protective effects from cytotoxicity were determined by MTT. Each value represents the mean ± SE of three replicate experiments, and the results are expressed as population growth (control as 100%). *: *p* < 0.05, **: *p* < 0.01 significantly different from at day 0 in same treated group. #: *p* < 0.05, ##: *p* < 0.01 significantly different from dd.H_2_O group at day 1 or day 3. dd.H_2_O: vehicle control; RS: rosiglitazone; LIOP: Low-molecular-weight, 10–100 kDa polysaccharides from *Inonotus obliquus* extract.

The viability of renal tubular cell (LLC-PK1) was used to determine whether LIOP confers protection against STZ + AGEs toxicity by MTT. Incubation of LLC-PK1cells with STZ (8 mM) and AGEs (3 mg/mL) resulted in a decrease in cell viability to 24% and 45% of controls (represented as percentage of the day 0) after 24 and 72 h of incubation, respectively (Figure 7). An incubation period of 24–72 h with LIOP (100 and 300 μg/mL) or RS (100 μg/mL), a marked increase in cell survival over those treated with dd.H_2_O alone. At 72 h, the cell viability of LIOP (100 and 300 μg/mL) were significantly dose dependently higher compared to that of dd.H_2_O alone (*p* < 0.05 and *p* < 0.01, respectively), suggesting that LIOP treatment might prevent from STZ + AGEs-induced glucotoxicity in renal cell.

### 2.8. The Effects of LIOP in the NF-κB and TGF-β Expression on Renal Tubular Cell

**Figure 8:**
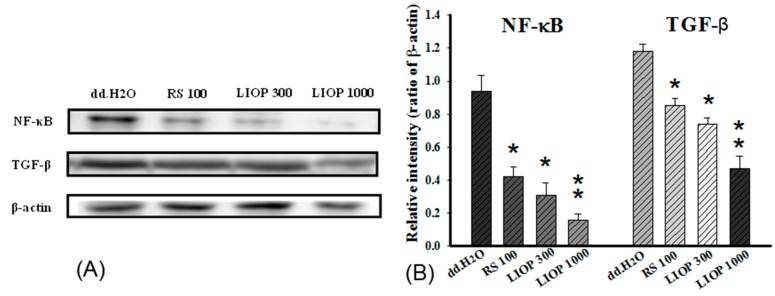
Fibrosis-associated proteins expression in renal tubular epithelial cells. The renal tubular epithelial cells (LLC-PK1) in vitro treated with STZ (8 mM) + AGEs (3 mg/mL) or co-treatment of LIOP (100 and 300 μg/mL) and RS (100 μg/mL) were collected after a three-day incubation. Expression levels of the cytoplasmic NF-κB, TGF-β1 and β-actin proteins were determined by Western blot and quantitated by microcomputer image device (MCID) image analysis (**A**). The β-actin levels were evaluated as a loading control, and the data are expressed as the NF-κB(p65)/β-actin and TGF-β/β-actin ratios, respectively (**B**). Each value represents the mean ± SE of three replicate experiments, and the results are expressed as population growth (control as 100%). *: *p* < 0.05, **: *p* < 0.01 significantly different from dd.H_2_O group. dd.H_2_O: vehicle control; RS: rosiglitazone; LIOP: Low-molecular-weight, 10–100 kDa polysaccharides from *Inonotus obliquus* extract.

In order to elucidate the anti-fibrosis effects of LIOP, the expression of nuclear factor-κB (NF-κB) and transforming growth factor beta (TGF-β) protein in renal tubular cell (LLC-PK1) were measured by Western blotting after three days of administration. A significant decrease in NF-κB (p65) and TGF-β expression was observed in the LIOP (100 and 300 μg/mL) and RS (100 μg/mL)-treated group compared with the dd.H_2_O group (*p* < 0.05, *p* < 0.01 and *p* < 0.05, respectively) (Figure 8: left panel). We also found that LIOP had dramatically decreased the levels of NF-κB and TGF-β expression in a dose-dependent manner and the inhibition (by ratio of β-actin) was noticeably higher in LIOP (300 μg/mL) treatment than in RS (100 μg/mL) treatment (Figure 8: right panel).

## 3. Discussion

In this study, the effects of the low-molecular-weight polysaccharides of *Inonotus obliquus* (LIOP) in high-fat diet (HFD) plus streptozotocin (STZ)-induced type 2-like diabetic nephropathy C57BL/6 mice were evaluated. The results demonstrated that eight weeks of administration of 10–100 kDa, LIOP (300 mg/kg) showed marked hypoglycemic, hypolipidemic and reno-protective effects in diabetic nephropathy mice in vivo. As supportive in vitro evidence, indicating LIOP (100 μg/mL) at incubation periods of 72 h also prevents STZ + AGEs (Streptozotocin + Advanced glycation end products)-induced glucotoxicity by decreasing cytoplasmic NF-κB and TGF-β expression in LLC-PK1 cells. These findings illustrated that 10–100 kDa (*M*w), LIOP could profoundly improve glucolipotoxicity-induced kidney dysfunction and sequential nephrofibrosis in diabetic nephropathy mice.

Chronic kidney disease (CKD), also known as chronic renal disease, is a major complication of metabolic syndromes including obesity, hypertension and diabetes mellitus. Comorbidity of the symptom is the risk factor for exacerbating progression of CKD. A previous report indicated that type 2 diabetes-induced kidney injury can mainly be attributed to the attenuation of dyslipidemia and insulin resistance, and ensuing oxidative stress and renal inflammation [31]. STZ is a selective cytotoxic action upon pancreatic beta-cell, leading to defective glucose oxidation causing hyperglycemia in the experimental diabetic model [32]. Once mice were fed with a high-fat diet (HFD), followed by a single intraperitoneal injection of STZ, an experimental diabetic nephropathy (DN) model was established [33]. *Inonotus obliquus* polysaccharides (IOPs) have therapeutic effects, such as anti-oxidant anti-inflammatory, anti-tumor and immunomodulating effects [34,35,36].

Besides, molecular weights of IOPs also influence their biological activities; particularly the low-molecular-weight IOP exerted more potent biological properties, including anti-oxidant, immune-modulatory and anti-inflammatory effects [37,38]. Thus, the hypoglycemic and lipid-lowering effects of the low-molecular-weight IOP, LIOP (10–100 kDa, *M*w) in diabetic nephropathy mice was conducted for evaluating its nephroprotective effects in the present study. The dry matter of culture broth (DMCB) of IO is capable of reducing glucose, triglycerides, fat acids, and cholesterol levels in blood and nearly normalized lipid profile after three weeks, eventually causing a regeneration of pancreatic tissue in alloxane-induced diabetic mice [39]. Even more, PIO is effective in the protection of STZ-induced diabetic rats [40]. In this study, our data is in agreement with previous hypoglycemic reports; the sensitivity to glucose weas progressively increased by OGTT, and the insulin tolerance were gradually reduced by ITT, respectively, in the LIOP groups (300 and 1000 mg/kg) after an eight-week administration. Several water extracts from IO improved insulin sensitivity and reduced adiposity in high fat (HF)-fed obese mice and rat [41,42]. Ethyl acetate fraction from *Inonotus obliquus* (EAFI) showed significant anti-hyperglycaemic and anti-lipidperoxidative effects in alloxan-induced diabetic mice [43]. Accordingly, we also found that LIOP groups (300 and 1000 mg/kg) reduced triglyceride levels and elevated the HDL/LDL on diabetic nephropathy mice in a time-dependent manner.

Medicinal mushrooms, *Ganoderma lucidum* polysaccharides (GL-PS), were able to reduce the serum creatinine (Cr), blood urea nitrogen (BUN) levels and urinary albumin excretion (UAE) in a dose-dependent manner in diabetic mice [44]. The changes in BUN and Cr revealed that *Cordyceps sinensis* polysaccharide (CPS-2) could significantly relieve renal failure caused by fulgerizing kidney [45]. However, there has not been until now information about the effects of IOPs on retarding renal fibrosis in type 2-like diabetic nephropathy in a mice model. It is noteworthy that urinary ACR were significantly declined in the high-dose LIOP (1000 mg/kg) group as with the RS (10 mg/kg) group at four and eight weeks. Although there was no significant difference in urinary ACR between the LIOP (300 and 1000 mg/kg) and RS (rosiglitazone, 10 mg/kg) groups (Figure 5B), urinary ACR was significantly decreased in LIOP (1000 mg/kg)-treated and RS (rosiglitazone, 10 mg/kg)-treated C57BL/6 mice compared with saline-treated C57BL/6 mice at four weeks and eight weeks. Accordingly, LIOP (100 μg/mL) incubation also prevent STZ + AGEs-induced glucotoxicity in renal tubular cells (LLC-PK1) in a time-dependent manner (Figure 7). Consistently, the reno-protective effect of LIOP is also evident from the histopathological examination on integrity of the glomerular capsules and the numbers of glomerular mesangial cells. The microscopic examination revealed that LIOP (1000 mg/kg) as well as RS (10 mg/kg) can significantly attenuate abnormal segmental expansion of the mesangium and mesangial matrix accumulation in renal cortex. In cultured glomerular mesangial cells (GMCs), the markedly increased NF-κB-regulated inflammatory factors TGF-β1 were consistent with the decrease of cell proliferation in high glucose-treated GMCs, leading to renal fibrosis [46]. Interestingly, our data also showed that the glomerular TGF-β1 protein expression was significantly decreased in LIOP (1000 mg/kg) rather than RS (10 mg/kg) in diabetic nephropathy mice. These results strongly suggest the great potential of the LIOP as anti-fibrotic through the inhibition of TGF-β during chronic kidney disease.

Renal insufficiency often markedly increases the circulating advanced glycation end product (AGEs) concentrations; therefore, AGEs are deemed to play a key factor during pathogenesis of diabetic nephropathy (DN) with diabetes complications [47]. Nevertheless, many investigators having focused on the glomerulus as a major target of the occurrence of DN. However, the major features of DN also included tubulointerstitial inflammation and tubular injury. The findings suggested that the changes in tubulointerstitial injuries are closely related to the amount of albuminuria and renal dysfunction [48]. A relational study also indicated that renal tubular functional and morphological changes appeared prior to the occurrence of microalbuminuria in the early stages of DN [49]. As mentioned previously, the role of tubulointerstitial injury cannot be excluded in the progression of DN. A related report also indicated that the progression of diabetic nephropathy may be attributed partially to the apoptosis of tubular epithelial cells (TECs), which causes hyperglycemia from oxidant stress accompanied with massive free radicals [50].

In our in vitro data, 72 h cell viability was significantly increased with LIOP (100 and 300 μg/mL) incubation in a dose-dependent manner, suggesting that LIOP treatment might prevent from STZ + AGEs-induced glucotoxicity in renal tubular cell (LLC-PK1). A recent study indicated that hyperglycemia induced NF-κB activation leading to an increase in inflammatory cytokines, IL-1β and TNF-α level, in diabetic kidneys [51] and an increase in TGF-β1 gene expression in STZ-induced diabetic rats [52]. Tubulointerstitial renal fibrosis, characterized as an accumulation of extracellular matrix protein, leads to chronic kidney diseases (CKDs) by upregulating TGF-β1 signaling pathway in STZ-induced diabetic rats [53]. Our results further supported that a dramatic decrease in NF-κB (p65) and TGF-β expression was observed in the LIOP treatment in renal tubular cell (LLC-PK1) in a dose-dependent manner. These results suggest that LIOP reduces renal fibrosis in diabetic nephropathy possibly through the NF-κB and TGF-β1 signaling pathways.

Taken together, low-molecular-weight polysaccharides of *Inonotus obliquus* (LIOP) may be advantageous to control the progression of CKD in mice with comorbid metabolic disorders. Moreover, particularly, the anti-glucolipotoxic and anti-nephrotoxic effects of LIOP accompanied by inhibition of NF-κB and TGF-β1 activation might be helpful mechanisms to reduce tubulointerstitial renal inflammation and fibrosis in diabetic nephropathy.

## 4. Materials and Methods

### 4.1. Chemicals and Reagents

Culture medium RPMI-1640, fetal bovine serum, sodium bicarbonate, l-glutamine, and 0.05% trypsin-EDTA were from Gibco Ltd. Streptozotocin (STZ) and nicotinamide (NA) were from Sigma (Saint Louis, MO, USA). Rosiglitazone maleate was from S.B. Pharmco Puerto Rico Inc. (Cidra, Puerto Rico, Certenejas). The rabbit polyclonal antibodies-TGF-β, HRP-anti-rabbit IgG and HRP anti-mouse IgG were from Santa Cruz Biotechnology, Inc. (Delaware, CA, USA). The anti-NF-κB p65 were purchased from Cell Signaling Technology (Danvers, MA, USA). Mouse insulin ELISA kit was from Mercodia (Sylveniusgatan, Uppsala, Sweden).

### 4.2. Preparations for Polysaccharide Extracts of Inonotus obliquus

The commercial fruiting body of *Inonotus obliquus* (IO) used in this study was purchased from the Taipei traditional Chinese medicine market (Taipei, Taiwan), and the pulverized IO was extracted (water to raw material ratio: 50 mL/g) through ultrasonic-assisted extraction at 100 °C for 1.5 h (frequency of 40 kHz). The water-soluble polysaccharide of IO (IOP) was precipitated by addition of 95% EtOH (*v*/*v*) for 48 h, and the filtrate was followed by protein depletion. Finally, the extract was concentrated by using a rotary evaporator, and then powdered by using a freezing-drying system (Kingmech, FD 20L-6S, Taipei, Taiwan)). The total polysaccharide yield was determined by the method of the phenol-sulphuric acid method using glucose as the standard reference.

### 4.3. Molecular Weight Determination by GPC

The molecular weight distribution of crude IOP was mainly determined by Gel Permeation Chromatography (GPC, SB-804 HQ column) and the molecular weight cut-off (30, 50 and 100 kDa) values were obtained by ultrafiltration spin columns. The tested sample was dissolved in distilled water at a concentration of 5 mg/mL, and 20 mL of this solution was injected. The column was operated at 45 °C and eluted with distilled water at a flow rate of 0.6 mL/min. The molecular weight estimation of polysaccharide was calibrated with series of dextran T10, T40, T70, T500 as molecular standards using the method of Yang’s with a slight modification [54]. The molecular weight of each fraction was obtained from the regression line of the standard molecular weight versus elution volume plot. Additionally, the crude IOP was transferred in the disposable ultrafiltration device (Vivaspin^®^ 500, Polyethersulfone) and was spun at 4000× *g* for 1 h at 20 °C for collecting the low-molecular-weight, 10–100 kDa polysaccharides (LIOP), from crude IOP. Finally, the filtered samples were dried in a vacuum centrifuge and stored at −80 °C until subsequent analyses.

### 4.4. Animal Preparation

The six-week-old male C57BL/6 mice were from National Laboratory Animal Center, Taipei, Taiwan. All animals were maintained in laminar flow cabinets under specific pathogen-free (SPF) conditions in facilities approved for Accreditation of Laboratory Animal Care and in accordance with Institutional Animal Care and Use Committee (IACUC) of the Animal Research Committee in Chi-Mei Medical Center, Tainan, Taiwan (IACUC Approval No. 100-004). The mice were housed separately and maintained on a 12 h light/dark cycle and were given orally a high-fat diet consisting of 40% (wt/wt) fat, whereas the normal group received a normal diet (Standard Diets: 5001-Laboratory Rodent Diet (LabDiet^®^) for eight consecutive weeks. The mice were induced by intraperitoneal injection of 50 mg/kg of STZ (in citrate phosphate buffer) and 200 mg/kg of NA for seven consecutive days. Oral administration of LIOP (300 and 1000 mg/kg) were conducted in the mice for four and eight consecutive weeks and the effect of rosiglitazone (10 mg/kg) is relative to the treated group as a positive control. During the experiments, the body weight was also estimated and blood was withdrawn from the retro orbital sinus of animals each week for detecting fasting and non-fasting plasma glucose levels.

### 4.5 Oral Glucose Tolerance Test (OGTT)

After an overnight fast, 3 g/kg d-glucose was administered orally to high-fat diet + STZ-induced C57BL/6 mice treated with LIOP (300 and 1000 mg/kg) as compared with rosiglitazone (10 mg/kg) administration and vehicle control (saline). To measure the fasting blood glucose levels, the blood samples were collected from each subject at 0, 15, 45, 95, and 135 min relative to the start of the oral glucose administration. The blood glucose level was analyzed using MAJOR II (Taipei, Taiwan).

### 4.6. Insulin Tolerance Test (ITT)

After an overnight fast, 0.5 U insulin was intraperitoneally injected into high-fat diet + STZ-induced C57BL/6 mice treated with LIOP (300 and1000 mg/kg) as compared with rosiglitazone (10 mg/kg) administration and vehicle control (saline). To measure the fasting blood glucose levels, the blood samples were collected from each subject at 0, 15, 45, 95, and 135 min relative to the start of the oral glucose administration. The blood glucose level was analyzed using MAJOR II (Taipei, Taiwan).

### 4.7. Determination of Biochemistry Indexes

The regiments composed of (i) before high-fat diet feeding + STZ injection; (ii) eight weeks after the end of the high-fat diet feeding + STZ injection and the treatment of LIOP (300 and 1000 mg/kg) and rosiglitazone (10 mg/kg) were implemented for C57BL/6 mice, respectively. The blood samples were prepared and centrifuged, and then biochemistry indexes were detected individually. The plasma insulin was measured with an insulin ELISA kit (Mercodia AB, Uppsala, Sweden) and the plasma triglycerides, cholesterol, HDL, LDL and urine albumin-creatinine ratio, respectively, were measured by the HITAICHI 7050 biochemistry analyzers.

### 4.8. The Hematoxylin and Eosin (H & E)

After mice were sacrificed, the excised kidney samples were fixed in formalin. The samples were dehydrated through a gradient mixture of ethyl alcohol and water, then rinsed with xylene before being embedded in paraffin. The formalin fixed tissues were sliced by silane-coated slides using a Microtome RM2135 (Leica Microsystems Inc., Bannockburn, IL, USA) in 5 μm sections and immersed in Tris-buffered saline (TBS, pH 7.4) after being rehydrated in graded ethanol solutions, dried at 37 °C overnight, and then stored at room temperature. The 5 μm kidney sections were stained by hematoxylin (Shandon™ Gill™III) and Shandon Eosin Y (Thermo Scientific™). Lastly, the slides underwent microscopic examination by means of a Motic BA 400 microscope with Motic Advance 3.0 software (Motic Co., Fujian, China).

### 4.9. Immunohistochemical Stain

The kidney samples were fixed in formalin and dehydrated by a gradient mixture of ethyl alcohol and water. The samples were then rinsed with xylene and embedded in paraffin. The formalin fixed tissues were sliced by silane-coated slides using a Microtome RM2135 (Leica Microsystems Inc., Bannockburn, IL, USA) in 5 μm sections. The slides were immersed in Tris-buffered saline (TBS, pH 7.4) after being rehydrated in graded ethanol solutions, dried at 37 °C overnight, and then stored at room temperature. After that, the sections were soaked in 0.3% H_2_O_2_ to block the endogenous peroxidase activity, then placed in the 10 mM citrate buffer solution (pH = 6.0) and microwave boiled for 10 min for completing antigen retrieval. The sections were incubated with primary antibodies against TGF-β (1:250 dilution) in a humidified chamber at room temperature for 2 h. Then the LSAB2 detection and DAB substrate kits were used for staining processes according to the manufacturer DAKO’s instructions. Finally, the sections were counterstained with hematoxylin (Shandon™ Gill™III) and calculated by the number of nuclei per square millimeter using an eyepiece reticule. For the positive labeling index for TGF-β, each tissue slide was illustrated as an average percentage of dividing the numbers of a TGF-β-positive cell (visualized in brown) by the total numbers of nuclei (visualized in blue). With each staining run, both positive and negative controls were provided and an overexpression was considered positive if more than 10% of the cells were showing.

### 4.10. Anti-Glucotoxicity Assessment (in Vitro)

Renal tubular cells, LLC-PK1 were purchased from Food Industry Research and Development Institute (Hsin Chu, Taiwan) and were supplemented with RPMI-1640 medium supplemented with 10% fetal calf serum (FCS), 2 mM l-glutamine, 1.5 g/L sodium bicarbonate, 4.5 g/L glucose, 10 mM HEPES, and 1.0 mM sodium pyruvate. The 96-well flat-bottomed microtiter plates were maintained in a humidified atmosphere containing 95% air and 5% CO_2_ at 37 °C in an incubator. After the cells cultured in the exponential growth phase were treated with a combination of STZ (8 mM) and AGE (3 mg/mL), they were induced and treated with LIOP (100 and 300 μg/mL)/well. After day 1 and day 3 of incubation, the in vitro anti-proliferative effects of these compounds were determined by the MTT assay (at 570 nm) and the cell viability was expressed as a percentage of the control (dd.H_2_O group) cells (% of control).

### 4.11. Western Blotting Analysis

Western blotting was used to evaluate the p65 subunit of NF-κB and TGF-β expression on whole-cell lysate. In brief, cells were cultivated at 1.5 × 10^6^ LLC-PK1 cells per 100 mm dish. To obtain equal cell densities, LLC-PK1 cells were cultivated at 1.5 × 10^6^ cells per 100 mm dish after 48 h. After cultivation as mentioned above, the medium was removed from the culture plates, and cells were rinsed three times with PBS (phosphate-buffered saline) after having been incubated for 2 h. To get the cell lysate, the cells were placed on ice with a protease inhibitor cocktail and then the lysate was centrifuged at 15,000× *g* for 10 min at 4 °C. The total protein concentration was determined by bicinchoninic acid assay with Pierce™ BCA assay Kit (Thermo Fisher Scientific, Carlsbad, CA, USA) using bovine serum albumin as a standard. By electrophoretic separation, the proteins (30 μg) were revealed by blot according to standard procedures. Lastly, 6 μL 6× Laemmli buffer (Bio-Rad Laboratories, Inc., Munich, Germany) and protein lysate (30 μL containing 30 μg of total protein) were mixed then heated at 95 °C for 5 min. The protein samples separately contained 4% stacking gel and 10% resolving electrophoresis. First, 120 volts were used and after several minutes, 80 volts were used after the electrophoresis had finished removing the film to be placed in a gel sandwich (respectively textile fiber pad, filter paper, film, NC membrane). To carry on the protein move, the wet-type protein moved through full transfer buffer by 400 mA. After blocking, membranes were then incubated for 1 h at room temperature in wash buffer with either anti-NF-κB antibody (1:5000) or anti-TGF-β antibody (1:500), followed by four times of 10 min washing. Horseradish peroxidase-conjugated anti-rabbit and anti-mouse IgG antibody were diluted to 1:5000 in washed buffer and incubated with blots for 1 h at room temperature. For measuring immunoreactive expression of NF-κB and TGF-β proteins from kidney, HRP assumed the stain (Reagent A + Reagent B by 1:1 proportion) on the NC membrane at room temperature After 1 min, it is developed again using the cold light image analyzer (FUJIFILM LAS-3000).

### 4.12. Statistical Analysis

Each value was expressed as the means ± SEM and analyzed using Sigma Plot 10.0 software (SPSS Inc., Chicago, IL, USA) to compare mean values between groups in a one-way ANOVA and Tukey’s test. *: *p* < 0.05, **: *p* < 0.01 and #: *p* < 0.05, ##: *p* < 0.01, respectively.
